# Microbiota-governed microRNA-204 impairs endothelial function and blood pressure decline during inactivity in db/db mice

**DOI:** 10.1038/s41598-020-66786-0

**Published:** 2020-06-22

**Authors:** Ravinder Reddy Gaddam, Veronica Peotta Jacobsen, Young-Rae Kim, Mohanad Gabani, Julia S. Jacobs, Karishma Dhuri, Santosh Kumar, Modar Kassan, Qiuxia Li, Raman Bahal, Robert Roghair, Kaikobad Irani, Ajit Vikram

**Affiliations:** 1 Department of Internal Medicine, Carver College of Medicine University of Iowa, Iowa City, IA-52242 USA; 2 Department of Paediatrics, Carver College of Medicine University of Iowa, Iowa City, IA-52242 USA; 3 Department of Pharmaceutical Sciences, University of Connecticut, Connecticut, USA

**Keywords:** Cardiovascular biology, miRNAs

## Abstract

An impaired decline in blood pressure at rest is typical in people with diabetes, reflects endothelial dysfunction, and increases the risk of end-organ damage. Here we report that microRNA-204 (miR-204) promotes endothelial dysfunction and impairment in blood pressure decline during inactivity. We show that db/db mice overexpress miR-204 in the aorta, and its absence rescues endothelial dysfunction and impaired blood pressure decline during inactivity despite obesity. The vascular miR-204 is sensitive to microbiota, and microbial suppression reversibly decreases aortic miR-204 and improves endothelial function, while the endothelial function of mice lacking miR-204 remained indifferent to the microbial alterations. We also show that the circulating miR-122 regulates vascular miR-204 as miR-122 inhibition decreases miR-204 in endothelial cells and aorta. This study establishes that miR-204 impairs endothelial function, promotes impairment in blood pressure decline during rest, and opens avenues for miR-204 inhibition strategies against vascular dysfunction.

## Introduction

The incidence of impaired decline in blood pressure at rest [non-dipping hypertension, (ndHTN)] is common in the diabetes patients^1,2^ and is believed to reflect endothelial dysfunction^3,4^. Diabetic patients with ndHTN are at higher risk of developing end-organ damage such as heart/kidney failure^2,5,6^. In the last decade, intestinal microbes have emerged as a determinant of hypertension^7–10^. Experimental studies demonstrate that the microbial transplantation from hypertensive rat to a previously normotensive rat can transfer hypertensive phenotype^7,11^. Further, the absence of gut microbiota rescues mice from the Angiotensin-II induced hypertension, endothelial dysfunction, and end-organ damage^12^. Besides, the spontaneously hypertensive rats have distinct intestinal microbiota^10^, and microbial suppression by antibiotics can reduce blood pressure^10^. All these studies underline the role of intestinal microbiota in hypertension, though the molecular basis of host-microbiota interaction remains poorly understood.

The microRNAs (miRs) are non-coding RNAs that regulate the expression of target genes and are emerging as a mode of gut microbe-host molecular communications^13^. Intestinal microbiota-host interaction influences the circulating level of miRs^14^ and metabolites [e.g. tri-methylamine-N-oxide and short-chain fatty acids] that can contribute to the pathogenesis of CVDs^15,16^. Multiple studies establish the role of tri-methylamine-N-oxide and short-chain fatty acids^15,17,18^ in the CVDs, but the role of vascular miR as a potential mediator of microbial-influence on vascular function remains unexplored. We recently discovered that the microbiota-governed vascular microRNA-204 (miR-204) targets Sirt1 and impairs endothelial function, and systemic inhibition of miR-204 rescues endothelial dysfunction and vascular inflammation in the high-fat diet-fed mice^19^. MiR-204 is a species-conserved miR, is among the top 50 miRs in the vasculature^19^, and is known to play a role in the pulmonary arterial hypertension^20^ and smooth muscle cells calcification^21^. Besides, miR-204 is predicted to target several genes that play a crucial role in maintaining endothelial function by regulating the enzymatic nitric oxide production [e.g., Slc34a2^22^ & Sirt1^19^]. In connection with the mechanism through which intestinal microbiota regulates vascular miR-204, we previously provided evidence for the potential involvement of a serum-based factor^19^, though, we could not find the molecular connection. Several studies demonstrate that the circulating miR-122 is upregulated during obesity/diabetes^23–26^, and patients at risk of cardiovascular disorders (CVDs) have a higher circulating miR-122^27–31^. Further, the miR-122 can regulate Stat3 activation which is a known repressor of miR-204 expression^32^.

In this study, we test the hypothesis that miR-204 promotes endothelial dysfunction and impaired decline in blood pressure at rest during diabetes and investigate whether circulating miR-122 contributes to the vascular miR-204 upregulation.

## Results

### Vascular miR-204 is upregulated in db/db mice, and its absence rescues endothelial dysfunction

**Figure 1:**
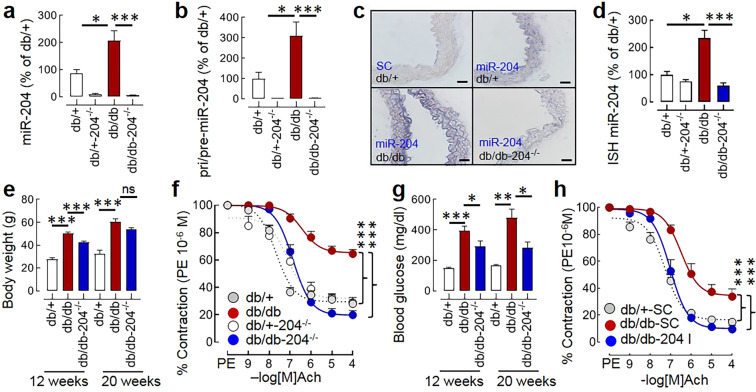
The miR-204 is upregulated in db/db mice and promotes endothelial dysfunction. (**a**) The aortic expression of mature miR-204 in the db/+, db/+−204^−/−^, db/db, and db/db-204^−/−^ mice. db/+: n = 8, db/+−204^−/−^: n = 4, db/db: n = 11, db/db-204^−/−^: n = 6. (**b**) The aortic expression of precursor (pri/pre) miR-204 in the db/+, db/+−204^−/−^, db/db, and db/db-204^−/−^ mice. db/+: n = 4, db/+−204^−/−^: n = 3, db/db: n = 6, db/db-204^−/−^: n = 6. (**c**) *In situ* hybridization (ISH) for miR-204 in aortas (blue, magnification ×40, Scale bar; 10 µm). (**d**) Quantification of aortic miR-204 in ‘c’. “n(N)” for ‘d’ where ‘n’ represents the number of images and ‘N’ represents the number of mice. db/+: n = 4(2), db/+−204^−/−^: n = 2(2), db/db: n = 8(2), db/db-204^−/−^: n = 5(2). (**e**) The body weight of db/+, db/+−204^−/−^, db/db, and db/db-204^−/−^ mice at the age 12 and 20 weeks (male ~50%). (**f**) Acetylcholine-mediated vascular relaxation of phenylephrine pre-contracted aortic rings isolated from db/+, db/+−204^−/−^, db/db, and db/db-204^−/−^ mice. The experimental replicate is shown as “n(N)” where ‘n’ represents the number of aortic rings and ‘N’ represents the number of mice. db/+: n = 13(4), db/+−204^−/−^: n = 11(3), db/db: n = 21(7), db/db-204^−/−^: n = 12(4). (**g**) The blood glucose level of db/+, db/+−204^−/−^, db/db, and db/db-204^−/−^ mice at the age of 12 and 20 weeks (male ~50%). (**h**) *Ex-vivo* inhibition of miR-204 rescues impairment of endothelium-dependent vasorelaxation in aortic rings isolated from db/db mice. The experimental replicate is shown as “n(N)” where ‘n’ represents the number of aortic rings and ‘N’ represents the number of mice. db/+−SC: n = 16(4), db/db-SC: n = 7(3), db/db-204 I: n = 6(3). A one-way analysis of variance (ANOVA) followed by Tukey’s test was performed. The significance of the difference between two curves was analyzed by using global non-linear regression. ns>0.05, *p < 0.05, **p < 0.01 and ***p < 0.001 vs. indicated group. The data are shown as mean, and the error bar represents s.e.m. Ach, acetylcholine; PE, phenylephrine.

The db/db mice spontaneously develop hyperphagia, hyperglycemia, and obesity with the advancement of age^33^. We found that the db/db mice overexpress miR-204 in the aorta (Fig. 1a). The determination of miR-204 precursors (pri/pre-miR-204) by qPCR and mature miR-204 in the histological sections of aorta *in-situ* hybridization (ISH) confirms the vascular upregulation of miR-204 in the db/db mice (Fig. 1b–d). To determine the role of miR-204 in diabetes-associated endothelial dysfunction, we generated db/db mice lacking miR-204 (db/db-204^−/−^, Suppl. Fig. 1a). The food and water intake of db/db-204^−/−^ mice were comparable to the db/db mice at 12 weeks and 20 weeks of age (Suppl. Fig. 1b,c), they were slightly leaner compared to db/db mice (at 12 weeks), but were obese compared to the db/+ mice (Fig. 1e). Next, we examined the acetylcholine (Ach) and sodium nitroprusside (SNP)-dependent vascular relaxation in the phenylephrine (PE, 10^−6^ M)-induced pre-contracted aortic rings to evaluate endothelium-dependent and endothelium-independent relaxation of the vascular rings, respectively. The aortic rings isolated from db/db mice had impaired endothelial function, while the endothelial function of the aortic rings isolated from db/db-204^−/−^ mice was significantly better (Fig. 1f). The aortic rings isolated from db/db mice also had slightly impaired endothelium-independent relaxation, and that was significantly better in the aortic rings isolated from db/db-204^−/−^ mice (Suppl. Fig. 1e). The db/db-204^−/−^ mice had a better glycaemic control (Fig. 1g), and that can *per se* improve endothelial function^34^. Thus, to determine the vascular role of miR-204, we inhibited miR-204 *ex vivo* in the aortic rings isolated from the db/db mice. The *ex vivo* inhibition of miR-204 resulted in a robust decline in aortic miR-204 (Suppl. Fig. 1d) and a significant improvement in the endothelium-dependent vascular relaxation (Fig. 1h). Interestingly, even in the *ex vivo* set-up the miR-204 inhibition improved endothelium-independent relaxation (Suppl. Fig. 1f).

**Figure 2:**
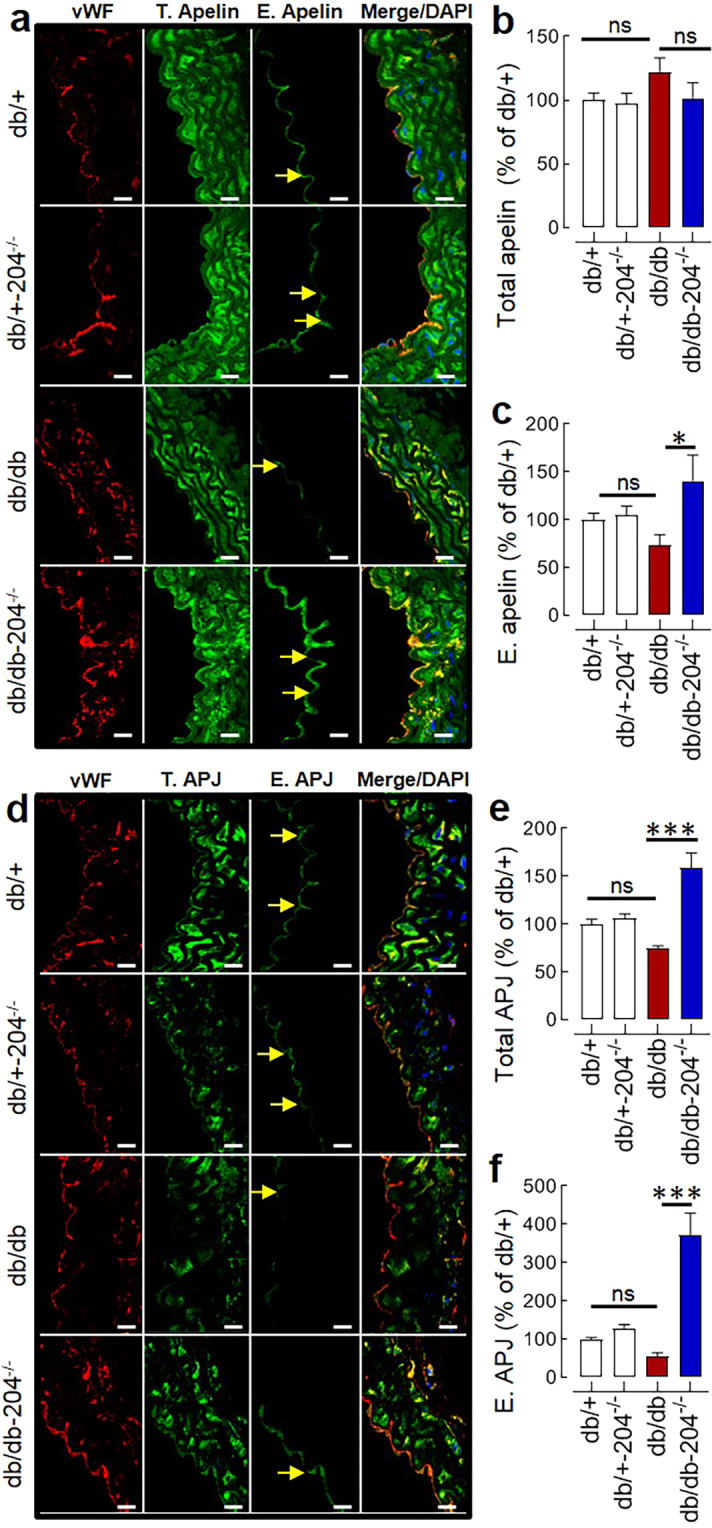
Endothelial expression of apelin and APJ in db/db & db/db-204^−/−^ mice. (**a**) Immunostaining of aortic sections showing total (T) and endothelial (E) apelin in db/db-204^−/−^ mice (magnification ×63, Scale bar; 10 µm). (**b,c**) Quantification of total (**b**) and endothelial (**c**) apelin in ‘a’. db/+: n = 10(4), db/+−204^−/−^: n = 10(4), db/db: n = 7(3) db/db-204^−/−^: n = 8(3). (**d**) Immunostaining of aortic sections showing total and endothelial APJ in db/db-204^−/−^ mice (magnification ×63, Scale bar; 10 µm). (**e,f**) Quantification of total (**e**) and endothelial (**f**) APJ in ‘d’. db/+: n = 14(4), db/+−204^−/−^: n = 14(4), db/db: n = 11(3), db/db-204^−/−^: n = 12(3). The experimental replicate is shown as “n(N)” where ‘n’ represents the number of images and ‘N’ represents the number of mice. ns>0.05, *p < 0.05, and ***p < 0.001 vs. indicated group. The data is shown as mean, and the error bar represents s.e.m. One-way analysis of variance (ANOVA) followed by Tukey’s test was performed. vWF, von-Willebrand factor.

A downregulation of apelin-receptor (APJ) has been reported in the aorta and renal arteries of the db/db mice, and exogenous apelin activates endothelial nitric oxide synthase in the db/db mice^35,36^. Thus, we examined whether the improved endothelial function in db/db-204^−/−^ mice is associated with an increased endothelial level of apelin and APJ. The apelin (mRNA and protein) in the aorta was not different between db/db and db/db-204^−/−^ mice (Fig. 2a,b, Suppl. Fig. 2a). Although we did not observe any difference in the APJ expression at the gene level (Suppl. Fig. 2b), the total APJ (protein) was higher in the aorta of db/db-204^−/−^ mice (Fig. 2d,e). As vascular endothelium constitutes a small part of the vasculature, the endothelial apelin and APJ was determined by co-immunostaining of the aortic sections with the endothelial marker von-Willebrand factor (vWF). The vascular endothelium of db/db-204^−/−^ mice had a significantly higher apelin and APJ compared to the db/db mice (Fig. 2c,f).

### MiR-204 is crucial for microbial effect on the diabetes-associated endothelial dysfunction

**Figure 3:**
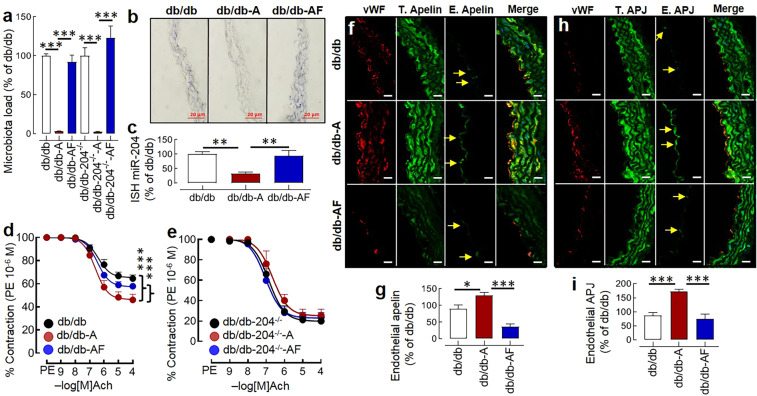
The microbial suppression decreases aortic miR-204 and improves endothelial function in db/db mice. (**a**) The fecal microbiota load in the db/db and db/db-204^−/−^ mice following antibiotics treatment (A) and microbiota recolonization (F). n = 5–10. (**b**) ISH showing aortic miR-204 following antibiotics-induced microbial suppression and recolonization (blue, magnification ×40, Scale bar; 20 µm). (**c**) Quantification of miR-204 expression in ‘b’. “n(N)” for ‘c’ where ‘n’ represents the number of images and ‘N’ represents the number of mice. db/db: n = 8(2), db/db-A: n = 8(2), db/db-AF: n = 8(2). (**d,e**) The acetylcholine mediated vascular relaxation of the aortic rings isolated from db/db and db/db-204^−/−^ mice after microbial suppression (A) and recolonization (F). The experimental replicate is shown as “n(N)” where ‘n’ represents the number of aortic rings and ‘N’ represents the number of mice. db/db: n = 21(7), db/db-A: n = 9(5), db/db-AF: n = 12(5), db/db-204^−/−^: n = 12(4), db/db-204^−/–-^A: n = 4(3), db/db-204^−/–-^AF: n = 14(6). (**f**) Immunostaining of aortic sections showing upregulation of endothelial apelin with antibiotics treatment and reversal with recolonization (magnification ×63, Scale bar; 10 µm). (**g**) Quantification of endothelial apelin in ‘f’. db/db: n = 9(2), db/db-A: n = 6(2), db/db-AF: n = 6(2). (**h**) Immunostaining of aortic sections showing upregulation of endothelial APJ with antibiotics treatment and reversal with recolonization (magnification ×63, Scale bar; 10 µm). (**i**) Quantification of endothelial APJ in ‘h’. db/db: n = 8(2), db/db-A: n = 8(2), db/db-AF: n = 8(2). The experimental replicate for ‘g’ and ‘i’ is shown as “n(N)” where ‘n’ represents the number of images and ‘N’ represents the number of mice. *p < 0.05, and ***p < 0.001 vs. indicated group. The data is shown as mean, and the error bar represents s.e.m. A one-way analysis of variance (ANOVA) followed by Tukey’s test was performed. The significance of difference between two curves was analyzed by using global non-linear regression. Ach, acetylcholine; PE, phenylephrine; vWF, von-Willebrand factor.

To test whether the intestinal microbiota-miR-204 axis is biologically relevant in diabetes-associated endothelial dysfunction, we provided antibiotics to the db/db and db/db-204^−/−^ mice, and a subgroup of antibiotics-treated db/db and db/db-204^−/−^ mice received fecal microbiota from the age-matched db/db mice. The microbial suppression in the antibiotics treated group was confirmed by the 16S RNA gene analysis in the freshly isolated fecal samples. Broad-spectrum antibiotics treatment led to over 95% decline in the fecal microbiota load and was restored by the recolonization (Fig. 3a). The antibiotics treated db/db mice had approximately 50% decline in the aortic miR-204 expression (Fig. 3b,c), and a significant improvement in the endothelial function (Fig. 3d). The antibiotics treated db/db mice in which microbes were recolonized had higher vascular miR-204 (Fig. 3b,c) and an impaired endothelium-dependent vascular relaxation (Fig. 3d). In contrast, the db/db-204^−/−^ mice had a better endothelial function (at baseline), which was neither improved by antibiotics treatment nor deteriorated by microbial recolonization (Fig. 3e) despite a comparable change in microbiota in response to antibiotics and recolonization (Fig. 3a). We did not observe any significant difference in the body weight and blood glucose level in the antibiotics-treated or recolonized db/db or db/db-204^−/−^ mice (Suppl. Fig. 3a,b). The aorta of antibiotics-treated db/db mice had better endothelium-independent relaxation compared to db/db mice (Suppl. Fig. 1g). The endothelium-independent relaxation of db/db-204^−/−^ mice was neither improved by antibiotics treatment nor deteriorated by the microbial recolonization (Suppl. Fig. 1h). As we observed that the db/db-204^−/−^ mice had higher expression of apelin and APJ in the endothelium, we asked if microbial suppression and recolonization also affect the apelin and APJ expression. We found that the microbial suppression led to a significant increase in the endothelial apelin and APJ expression, which reversed with the microbial recolonization (Fig. 3f-i).

### Circulating miR-122 is upregulated in db/db mice, and its systemic inhibition decreases vascular miR-204

**Figure 4:**
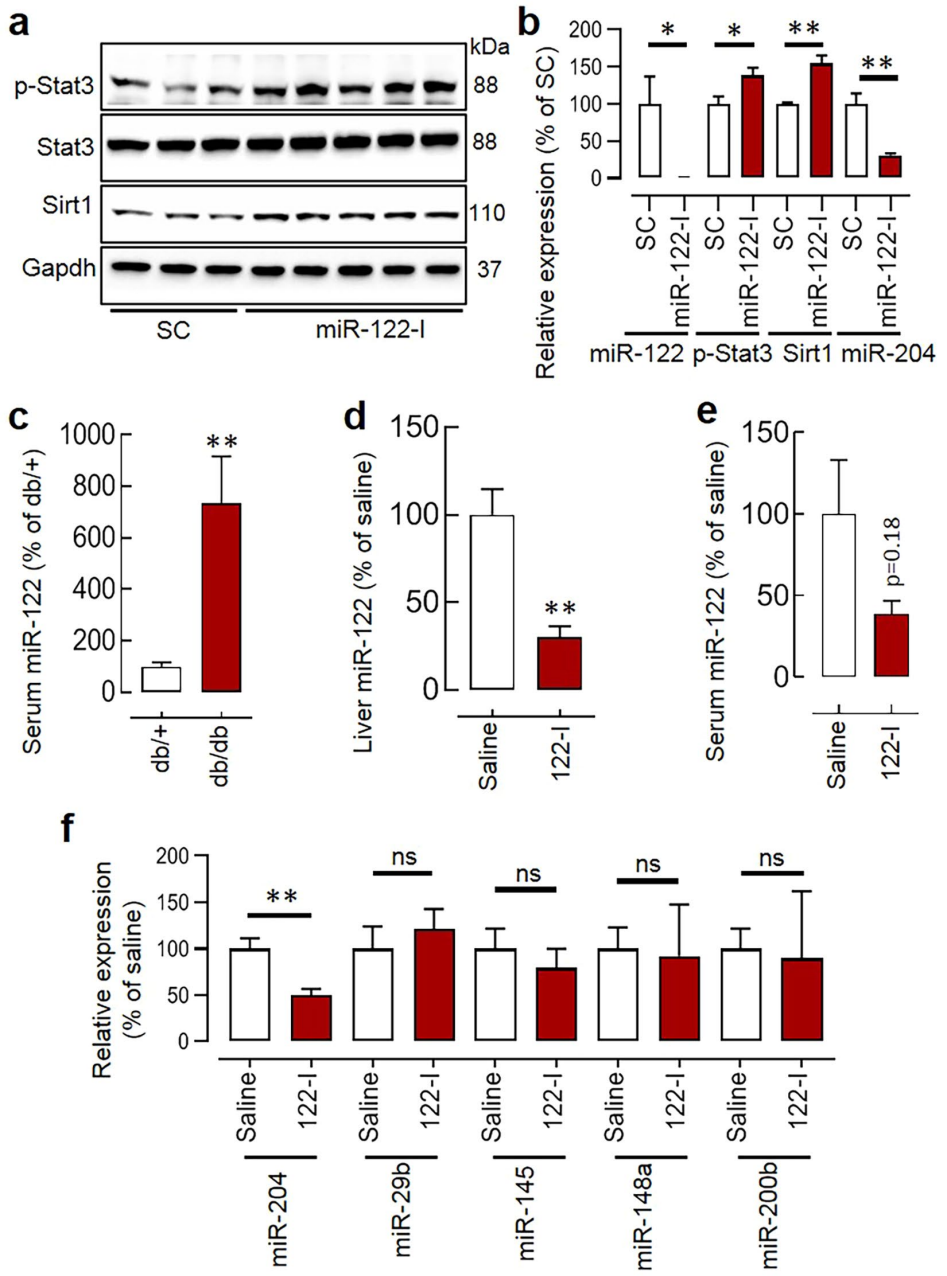
The miR-122 regulates miR-204 expression: (**a,b**) Silencing of miR-122 (miR-122 I; 20 nM) in Human Umbilical Vein Endothelial Cells (HUVECs) increased Stat3 phosphorylation (Tyr-705), decreased miR-204 expression, and increased expression of miR-204 target gene Sirt1. n = 3–6. The full-length blots are presented in Supp. Fig. S6. (**c**) The circulating miR-122 in the db/db mice. n = 14–15. (**d,e**) Systemic silencing of miR-122 by PNA-122-I led to a significant decrease in hepatic miR-122 (**d**) and a tendency showing decreased serum miR-122 (**e**). (**f**) Expression of miR-204 and other common vascular miRs such as miR-29b, miR-145, miR-148a, and miR-200b in the aorta of db/db mice receiving either saline or PNA-122-I. n = 3–4 in all groups. *p < 0.05, and **p < 0.01 vs. indicated group. The data are shown as mean, and the error bar represents s.e.m. An independent sample t-test was used.

Circulating miR-122 is upregulated during obesity/diabetes^23–26^, patients at risk of CVDs have a higher circulating miR-122^27–31^, and miR-122 can regulate Stat3 activation which is a known repressor of miR-204 expression^32^. Further, the antibiotics-treated wildtype and db/db mice have lower circulating miR-122 (Suppl. Fig. 4a,b). Therefore, we investigated the potential involvement of miR-122 in the regulation of vascular miR-204. In our *in-vitro* studies in Human Umbilical Vein Endothelial Cells (HUVECs), the miR-122 inhibition increased Stat3 phosphorylation, decreased miR-204 expression, and led to an increase in the expression of miR-204 target gene Sirt1 (Fig. 4a,b). We inquired about the circulating level of miR-122 in the db/db mice, and it was significantly higher (Fig. 4c). The next logical step was to determine whether *in-vivo* inhibition of miR-122 can rescue vascular upregulation of miR-204 in the db/db mice. Therefore, we designed and synthesized the novel peptide nucleic acid (PNA)-based miR-122 inhibitor (PNA-122-I). PNAs are a synthetic DNA mimic that has charge-neutral N-(2-aminoethyl) glycine backbone instead of phosphodiester backbone that makes it efficient and resistant to the proteases/nucleases. PNAs can bind single-strand targets with high specificity/affinity, and their charge-neutral attributes make them translationally attractive. We found that the PNA-122-I with 3 lysines on both the 3′ and 5′ end had better binding with miR-122 in physiological salt solution compared to the one with single lysine at the 3′ end (Suppl. Fig. 4c) and was able to inhibit hepatic miR-122 in mice at 20 mgkg^−1^day^−1^ (Suppl. Fig. 4d). The systemic inhibition of miR-122 at 20 mgkg^−1^day^−1^ for seven days in the db/db mice led to a decrease in hepatic miR-122 (Fig. 4d), a tendency of decrease in the circulating miR-122 (Fig. 4e), and a significant decrease in the aortic miR-204 (Fig. 4f). We also measured the expression of other common vascular miRs such as miR-29b, miR-145, miR-148a, and miR-200b in the aorta of db/db mice receiving either saline or PNA-122-I and their level remained unaffected (Fig. 4f).

### The db/db mice lacking miR-204 have an improved decline in blood pressure during inactivity

**Figure 5:**
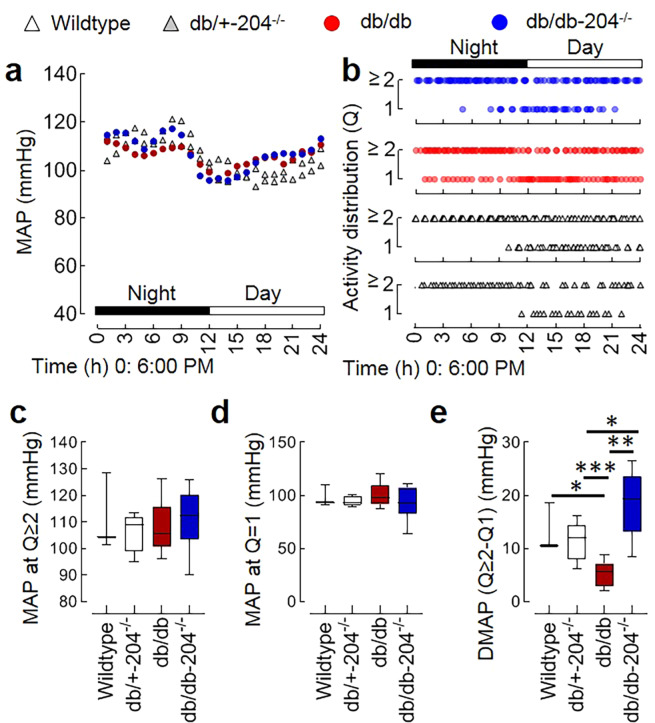
db/db mice lacking miR-204 have better blood pressure decline during inactivity. (**a**) The representative curve of 24 hr (day and night) telemetric recording of the average mean arterial pressure (MAP) in the wildtype, db/+−204^−/−^, db/db, and db/db-204^−/−^ mice. Wildtype: n = 3, db/+−204^−/−^: n = 7, db/db: n = 7, and db/db-204^−/−^: n = 6. (**b)** The mouse locomotor activity distribution in quartiles. The ‘y’ axis shows an hourly mean activity quartile. 1; 0–25%, ≥2; 25–100%. The ‘x’ axis shows time (24 h) beginning at 6:00 PM. (**c–e**) The MAP during activity quartile ≥2 (c), activity quartile 1 (**d**) and a difference in the MAP during activity quartile ≥2 and activity quartile 1 (**e**). Wildtype: n = 3, db/+−204^−/−^: n = 7, db/db: n = 7, db/db-204^−/−^: n = 6. *p < 0.05; **p < 0.01; ***p < 0.001 vs. indicated group. In box-and-whisker plots, whiskers show minima and maxima and the central line indicates median. The significance of the difference between groups was performed by one-way analysis of variance (ANOVA) followed by Tukey’s test.

We used 20 weeks old mice to test the role of miR-204 in the blood pressure decline during inactivity. Multiple cardiovascular parameters, including systolic arterial pressure, diastolic arterial pressure, heart rate, and mouse locomotor activity was simultaneously monitored at baseline as well as in the presence of nitric oxide synthase inhibitor (L-NAME) in the wildtype, db/+−204^−/−^, db/db, and db/db-204^−/−^ mice. As anticipated, mean arterial pressure (MAP) was higher during the night and lowered during the day in all groups (Fig. 5a). The MAP during day or night was not different among the wildtype, db/+−204^−/−^, db/db, and db/db-204^−/−^ mice (Suppl. Fig. 5a,b). The db/db mice had impaired circadian decline in the MAP, and the db/db-204^−/−^ mice did not differ from db/db mice in terms of a change in MAP (Suppl. Fig. 5c). The mice were generally inactive from 4:00 AM to 3:00 PM (Fig. 5b), so the traditional 12 hour light versus dark cycle epochs (6:00 AM to 6:00 PM versus 6:00 PM to 6:00 AM) would not accurately capture the decline in the MAP during inactivity (rest). Therefore, we contrasted the MAP during activity quartiles 2 to 4 (Q ≥ 2, 25–100%) against the MAP during activity quartile 1 (Q1, 0–25%). The MAP at activity quartile Q1 or activity quartile Q ≥ 2 was not different among wildtype, db/+−204^−/−^, db/db, and db/db-204^−/−^ mice (Fig. 5c,d). However, a decline in MAP during activity Q1 compared to MAP during activity Q ≥ 2 was significantly lower in the db/db mice compared to wildtype and db/+−204^−/−^ mice (Fig. 5e). The db/db-204^−/−^ mice had a significantly improved decline in the MAP during activity Q1 compared to MAP during activity Q ≥ 2 compared to the db/db mice (Fig. 5e). To understand the role of nitric oxide in the MAP decline, all the blood pressure parameters and mouse locomotor activity were recorded in the presence of L-NAME. The L-NAME treatment was associated with an increase in the MAP in all the groups (Suppl. Fig. 5d). The MAP during day and night was not different among the wildtype, db/+−204^−/−^, db/db, and db/db-204^−/−^ mice (Suppl. Fig. 5f,g). However, as we observed in the absence of L-NAME, the db/db mice had a significantly lower decline in the MAP during the day (Suppl. Fig. 5h). L-NAME treatment did not affect the activity cycle, and mice were inactive from 4:00 AM to 3:00 PM (Suppl. Fig. 5e). Although the MAP during activity Q1 was lower in all groups compared to the MAP during activity Q ≥ 2, we did not observe a significant difference in the MAP during activity Q ≥ 2 or activity Q1 among wildtype, db/+−204^−/−^, db/db, and db/db-204^−/−^ mice (Suppl. Fig. 5i,j). However, even in the presence of L-NAME, we observed an improved decline in MAP (Q ≥ 2 vs. Q1) in the db/db-204^−/−^ mice compared to db/db mice (Suppl. Fig. 5k).

## Discussion

One of the concerns in the attribution of ndHTN in the diabetes patients is increased nocturnal waking up (for reasons such as nocturia), which leads to the intermittent measurement of awake blood pressure and misclassification of patients as non-dippers. However, studies performed in patients at siesta (a short afternoon nap often not accompanied by the break) confirm the higher risk of ndHTN in T2D patients^37^. The Genome-Wide Association Studies (GWAS) have identified several genetic variants linked to hypertension, but cumulatively these variants explain less than 5% of the inter-individual variation in systolic blood pressure^38–40^. The GWAS studies highlight the importance of environmental factors such as diet and intestinal microbiota in the development of CVDs^9,41^. In this study, we investigated the role of miR-204 in vascular endothelial dysfunction and ndHTN during diabetes. Our results demonstrate that the absence of miR-204 rescues diabetes-associated endothelial dysfunction and impaired decline in the blood pressure at rest.

The db/db mice overexpress miR-204 in the vasculature, and its absence rescues endothelial dysfunction despite hyperphagia and obesity. Jo *et al*. show that the miR-204^−/−^ mice at baseline does not have any improvement in the glucose disposal^42^. Similarly, we also did not observe any difference in the glycaemic control between db/+-204^+/−^ and db/+-204^−/−^ mice (Suppl. Fig. 3c,d). However, the db/db-204^−/−^ mice had significantly better glycaemic control compared to the db/db mice. As miR-204 has previously been reported to play a role in pancreatic β-cell functions^32,42,43^, the absence of miR-204 might have improved β-cell function and hence the glycaemic control. Previous studies in db/db mice^44^ and humans^34,45^ have shown that a tighter glycemic control improves endothelial function. Thus, the improved endothelial function in db/db-204^−/−^ mice could be a secondary effect of the slightly lower body weight and a better glycemic control rather than the vascular absence of miR-204 or a combined effect of all. However, an improvement in the endothelial function with *ex vivo* miR-204 inhibition in aortic rings isolated from the db/db mice (Fig. 1h) suggests that vascular miR-204 contributes to the diabetes-associated endothelial dysfunction.

The vascular miR-204 is regulated by the intestinal microbiota^19^, and to determine the role of the microbiota-miR-204 axis in diabetes-associated endothelial dysfunction, we suppressed intestinal microbiota by antibiotics and recolonized them by stopping antibiotics and providing fecal microbiota from the donor (age-matched db/db) mice. The microbial suppression decreased aortic miR-204 and improved endothelial function in the db/db mice, and this effect was reversed with microbial recolonization. In contrast, the db/db-204^−/−^ mice had a better endothelial function at baseline, which neither improved with microbial suppression nor was impaired by microbial recolonization. Previous studies show that abnormal gut microbiota in db/db mice contributes to the development of diabetes in the db/db mice^46^. The fasting mimetic-diet changes microbial composition and survival of the db/db mice^47^. Further, the dietary supplements such as mulberry fruit polysaccharide, the polyphenol-rich extract of *Dendrobium Loddigesii*, and capsaicin change the intestinal microbial composition and improve glycaemic control in the db/db mice^48–50^. In our previous studies, we examined the effect of antibiotics in the normal chow-fed, and high-fat diet-fed mice where endothelial dysfunction is relatively milder, and a more extended antibiotics treatment were required. However, the db/db mice develop ~60% impairment in the endothelial function by 12 weeks of age, and a two-week antibiotics treatment was enough to improve the endothelium-dependent vascular relaxation. Further, the microbial suppression for two weeks and microbial recolonization did not have a significant effect on the body weight and blood glucose level in the db/db and db/db-204^−/−^ mice (Suppl. Fig. 3a,b). These data support that the protective effects of microbial suppression on endothelial function could be mediated through mechanisms other than improvement in the blood glucose level. The insensitivity of the endothelial function of db/db-204^−/−^ mice to microbial changes suggests that the presence of miR-204 is critical for the microbial effects.

The in-silico prediction tools at microrna.org for miR-mRNA interactions predicts that the mouse apelin (*apln*) is a weak putative target of miR-204. However, we did not see any significant difference in the apelin expression in the aorta at the gene as well as protein level between wildtype and miR-204^−/−^ mice (Fig. 2b, Suppl. Fig. 2a). There are a couple of studies that report the downregulation of APJ in the vasculature of db/db mice. They also found that the exogenous apelin activates Akt, endothelial nitric oxide synthase, and improves the endothelial function of the aorta of db/db mice^35,36^. The apelin is also known to lower blood pressure through a nitric oxide-dependent mechanism and have angiogenic effects^51,52^. Our results demonstrate that the endothelial expression of apelin and APJ was significantly higher in db/db-204^−/−^ mice compared to db/db mice. Similarly, both apelin and APJ were significantly higher in the vascular endothelium of db/db mice receiving antibiotics, which was reversed by microbial recolonization. These data suggest that intestinal microbes and diabetes both upregulate vascular miR-204, decrease endothelial apelin, and impair endothelial function.

MiRs are known to regulate the expression of other miRs^27,53^. miR-122 silencing is reported to decrease SOCS3 (suppressor of cytokine signaling 3) via promoter methylation^54^, and SOCS3 is a potent inhibitor of Stat3 phosphorylation (Tyr-705), which in turn is a repressor of miR-204^32^. In addition, multiple independent studies establish that the circulating miR-122 is upregulated during obesity/diabetes^23–26^, miR-122 promotes inflammation^55–58^, and the patients at risk of CVDs have a higher circulating miR-122^27–31^. Thus, anticipating that miR-122 is the potential regulator of vascular miR-204 we examined circulating miR-122 in the db/db mice, and it was significantly higher. In both *in-vitro* as well as *in-vivo* experiments we found that miR-122 regulates endothelial/vascular miR-204 expression. The miR-122 is a conserved liver-specific miR in vertebrates and circulating miR-122 has emerged as a sensitive biomarker of hepatic injury^59–62^. Perhaps, the increased circulating miR-122 during diabetes upholds the multiple pathological pathways in the vasculature, and miR-204 upregulation is just one of them.

The endothelial dysfunction in diabetes precedes and predicts ndHTN^3,4,63,64^. However, the pathogenesis of ndHTN is affected by multiple organ systems, is tightly regulated, and develops at the later stages of diabetes. Our finding that the db/db mice do not have a higher MAP is consistent with the previous report^65,66^ but differs from^67,68^. In the 12-h light/dark cycle we observed impairment in decline in MAP during the day in the db/db mice, but the db/db-204^−/−^ mice were not significantly different from the db/db mice. Our results showing that the db/db mice had an impaired circadian decline in MAP agree with previous reports^68,69^. The active and inactive phase of mice does not overlap with the 6:00 AM/6:00 PM light/dark cycle. The db/db mice are obese and are known to have decreased locomotor activity^68^, we also observed that the inactive phase was distributed across the 24 hrs. Therefore, we contrasted the MAP change with respect to the mouse locomotor activity and found that the db/db mice have a significant impairment in the MAP decline during inactivity, while the db/db-204^−/−^ mice have significantly better MAP decline during inactivity. An improved endothelial function of db/db-204^−/−^ mice might have contributed to a higher decline in the MAP during inactivity. Conversely, a shorter exposure of db/db-204^−/−^ mice to a higher blood pressure could also improve endothelial function. Interestingly, an improved MAP decline during inactivity in the db/db-204^−/−^ mice, even in the presence of L-NAME suggests that the absence of miR-204 improves MAP decline at rest through nitric-oxide independent mechanisms as well. Though the underlying molecular mechanisms remain elusive these results demonstrate that the absence of miR-204 improves MAP decline during inactivity in the diabetic mice.

One of the limitations of this study is that whether intestinal microbes regulate circulating miR-122, and if yes how, remains unknown. Although we demonstrate that the miR-122 regulates vascular miR-204, it is not clear whether miR-122 deregulation is the primary mechanism through which intestinal microbes regulate vascular miR-204. The additional experiments determining the effects of miR-204/miR-122 inhibition and that of microbial suppression/recolonization on the MAP decline during activity and inactivity would have further ascertained the microbial role on ndHTN through miR-204/miR-122 dependent mechanisms. In the replenishment/transplant study, both db/db and db/db-miR-204^−/−^ mice received microbiome from the db/db mice. The idea was to determine whether the microbiome from db/db mice can impair endothelial function in both db/db and db/db-204^−/−^ mice. However, we recognize that an alternative approach could be to transplant microbiota from the db/db-miR-204^−/−^ mice to the antibiotics-treated db/db and db/db-miR-204^−/−^ mice which will test whether an absence of miR-204 affects the intestinal microbial composition which in turn improves the glycaemic control and endothelial function. We also recognize that the present set of data is generated in the global miR-204^−/−^ mice which fail to discern whether the beneficial effects of miR-204 absence are of vascular and extravascular origin. The general downregulation of miR-204 might have a deleterious effect in a specific condition, and therefore, additional studies in the tissue-specific miR-204 knockout mice are warranted. Although multiple independent studies demonstrate miR-122 upregulation in patients with cardiovascular disorders^27,29,70,71^, the miR-122’s role in blood pressure regulation remains unknown. Based on our data, we anticipate that the db/db mice lacking miR-122 will have an improved vascular function and blood pressure dipping during inactivity. However, this needs to be experimentally verified.

The miR-204’s expression is regulated by intestinal microbiota and its absence rescues impaired blood pressure decline, which raises the potential effect of antibiotics on the blood pressure regulation. In experimental studies, the use of minocycline for four weeks was able to decrease the MAP in the Angiotensin-II infused rats^10^. The microbes are known to regulate the intestinal production of short-chain fatty acids (SCFAs) which are known to regulate the blood pressure through olfactory receptor Olfr78^72^. In a prospective study involving over 380,000 participants followed up for nine years the fiber intake, which is known to change the intestinal microbial demographics, was found to be associated with lower risk of cardiovascular disorders^41^. The dietary fiber is believed to act as prebiotics as they feed commensal bacterias and several epidemiological studies have shown that a high intake of fiber is associated with a reduced blood pressure^73,74^. The bacterial infection during pregnancy, as assessed by self-reported antibiotic usage, was associated with small rises in the blood pressure^75^. However, there is a paucity on the data regarding the use of antibiotics and probiotics in the context of an effect on microbial composition and blood pressure regulation. The present study does support the role of miR-204 in the diabetes-associated blood pressure deregulation, one of the limitation is that whether antibiotics mediates its effect on blood pressure through miR-204 remains unknown, and further study is warranted.

**Figure 6:**
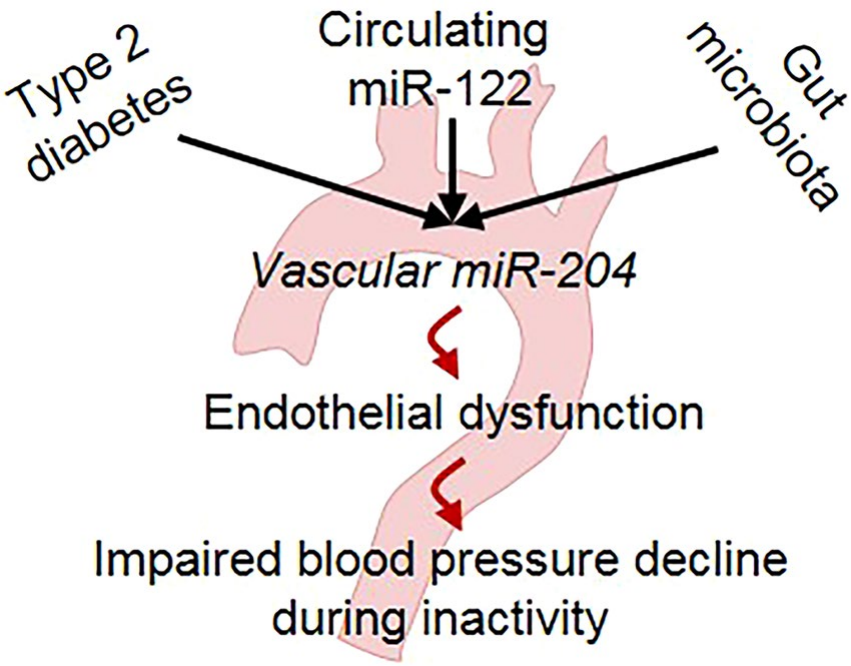
Schematic showing that diabetes, circulating miR-122, and intestinal microbiota promote vascular miR-204 expression, which in-turn promotes endothelial dysfunction and an impaired decline in blood pressure during inactivity.

Multiple studies demonstrate the beneficial effects of microbial demographics-altering strategies on cardiovascular outcomes, and miRs are emerging as a molecular means of host-microbiota communication. The present set of data demonstrates that the vascular miR-204 and circulating miR-122 are upregulated during diabetes, absence of miR-204 rescues endothelial dysfunction and impaired decline MAP during inactivity, and circulating miR-122 at least in part regulates vascular miR-204 expression (Fig. 6). Moreover, the inhibition of miR-204 represents a therapeutically attractive approach for rescuing diabetes-associated vascular dysfunctions.

## Methods

### Animals

All animal experiments were approved by the Institutional Animal Care and Use Committee of the University of Iowa and were carried out according to the National Institute of Health (NIH) guidelines. All the studies were performed in C57BL/6, miR-204 knockout (miR-204^−/−^), leptin-receptor mutant (db/db) and db/db-miR-204^−/−^ (db/db-204^−/−^) mice (age 10–22 weeks) of both sexes. All the animals received a standard rodent diet. Intestinal microbiota was suppressed with a cocktail of broad-spectrum antibiotics (50 mg.kg^−1^, vancomycin, 100 mg.kg^−1^ metronidazole, 100 mg.kg^−1^ ampicillin, and 100 mg.kg^−1^ neomycin) as previously described^19,76^. The antibiotic cocktail was administered to mice daily for 2 weeks by oral gavage (100 µL per 25 g mouse), using a stainless-steel feeding tube without prior sedation of the animals. For FMT, the fecal microbiota from the donor mice was transferred to recipient mice following antibiotic cocktail treatment. The fresh stool was collected every day from the db/db mice immediately upon defecation and was resuspended (1:10) in a sterile PBS solution containing 0.05% cysteine HCl. The sample was vortexed and then centrifuged at low speed (800 × g) for 2 min. A 200μl aliquot of the supernatant was orally gavaged to the recipient mice every day for two weeks. Mice were maintained in specific pathogen-free conditions at a central animal facility of the University of Iowa. The mice receiving a cocktail of antibiotics were monitored for weight loss, diarrhea and signs of discomfort. Suppression of gut microbiota was confirmed by bacterial 16S RNA gene analysis.

### Cell culture

Human umbilical vein endothelial cells (HUVECs, Cat# CC-2519) were purchased from Lonza (Mapleton, IL USA) and cultured in growth factor supplemented EGM-2 (Lonza, Walkersville, MD USA).

### Vascular reactivity

The vascular reactivity was determined as previously described^19^. Briefly, the thoracic aorta was carefully dissected, rapidly removed and placed in ice-cold oxygenated Krebs-Ringer bicarbonate solution. The vessels were cleaned of fat and loose connective tissue and cut transversely into 5–10 rings (1.5–2.0 mm wide). The rings were placed in oxygenated organ chambers (95% O_2_/5% CO_2_) filled with 6 mL of Krebs-Ringer buffer solution and maintained at 37 °C and pH 7.4. Each ring was suspended between two wire stirrups (150 µm) in 6 mL chambers of a myograph system (DMT Instruments, FL, USA). One stirrup was connected to a three-dimensional micromanipulator, and other to a force transducer. The mechanical force signal was amplified, digitalized and recorded (PowerLab 8/30). All concentration-effect curves were performed on arterial rings beginning at their optimum resting tone. This was determined by stretching arterial rings at 10 min intervals in an increment of 100 mg to reach optimal tone (~500 mg). One dose of KCl (60 mM) was administered to verify vascular smooth muscle viability. Endothelium-dependent and -independent vasorelaxation was determined by generating dose-response curves to acetylcholine (Ach,10^−9^–10^−4^ M) and sodium nitroprusside (SNP,10^−9^–10^−4^ M), respectively, on PE (10^−6^ M)-induced pre-contracted vessels. Vasorelaxation evoked by acetylcholine and sodium nitroprusside was expressed as percent relaxation, determined by calculating the percentage of inhibition to the pre-contracted tension. Aortic rings not responding to the initial dose of KCl or showing auto-relaxation were excluded.

### Transfection of oligonucleotides

The freshly isolated thoracic aortic rings from db/+ and db/db mice were transfected with oligonucleotides using Oligofectamine (Invitrogen). Oligos [miR-204 inhibitor and scrambled control (SC); 200 nM] were incubated with Oligofectamine (3 µL) at room temperature for 20 min before addition to the aorta in basal EGM (250 µL). The aortic rings were kept in the transfection mixture for 24 hrs (at 37 °C and 5% CO_2_). After 24 hrs of transfection, the media was removed, and the aortic rings were used to determine the vascular reactivity and expression of miR-204. Transfection of miR-122-I into the HUVECs was performed using lipofectamine2000 (Invitrogen). The sequence of oligonucleotides used in the study is provided in Suppl. Table 1.

### RNA isolation and qPCR

RNA was isolated using Qiazol/Trizol as per the manufacturer’s instructions. MicroRNAs and RNAs were converted to cDNA using the qScript microRNA cDNA synthesis kit (Quantabio). Real-time qPCR for miR-204, APLN, and APJ were performed using Brilliant II SYBR Green RT-qPCR kit. As no internal control is universally accepted for the miRs, we used a battery of internal controls for the quantification of miR in the aorta comprising Gapdh/18 s/RNU6. We observed the most consistent results with Gapdh. To quantify serum miR we used a constant amount of serum (200μl), and the Ct values were not normalized using any internal control. The Gapdh was used as an internal control for the qPCR-based quantification of ‘*Apln*’ and ‘*APJ*’ expression quantification. Primer sequences are provided in Suppl. Table 1.

### Total microbial load

Fecal bacterial DNA was isolated using the DNeasy PowerSoil Kit (Qiagen). The amount of DNA in each sample was quantified using nanodrop. Based on the nanodrop readings, each aliquot of the DNA sample was diluted to 1 ng/µL. To determine the microbial load, real-time qPCR for bacterial 16S RNA was performed using the Brilliant II SYBR Green RT-qPCR kit. Primer sequences are provided in Suppl. Table 1.

### Histological processing and immunostaining

Sections (5 µM) of formalin-fixed paraffin-embedded tissues were heated (95 °C, 20 min) in citrate buffer (10 mM) for antigen retrieval, followed by incubation with primary antibodies. The working concentrations of anti-apelin (LSBio- LS-C149244), anti-APJ (Millipore-Sigma- ABD43), and vWF (Abcam-ab11713) antibodies for the immunofluorescence experiments were 1:100. Antigen-primary antibody complexes were probed with fluorescence-tagged secondary antibodies. Images were captured using a Zeiss confocal microscope (Model 710). To examine the change in endothelial expression of apelin and APJ the endothelial cells were cropped from the images based on the vWF staining using Photoshop CC software, and the ImageJ software was used to determine the intensity. In the intensity quantification analysis, the “n” represents the number of aortic images, while “N” represents the number of mice.

### Synthesis of PNA anti-miR-122

The PNA-based antimiR was synthesized as previously described with minor modifications^77^. Boc-protected PNA monomers used for PNA synthesis were purchased from ASM Chemicals and Research (Hanover, Germany). PNAs were synthesized via solid-phase synthesis using MBHA (4-Methylbenzhydrylamine) resin and Boc-monomers (A, T, C, G). PNAs were then cleaved from the resin using a cleavage cocktail containing m-cresol: thioanisole: trifluoromethanesulfonic acid (TFMSA): trifluoroacetic acid (TFA) (1:1:2:6) followed by precipitation using diethyl ether. PNAs were purified and characterized using reverse-phase high-performance liquid chromatography (HPLC) and matrix-assisted laser desorption/ionization-time of flight (MALDI-TOF) spectroscopy, respectively. The concentration of PNAs was determined using UV-vis spectroscopy, and the extinction coefficient of PNAs was calculated using the extinction coefficient of individual monomers (13,700 M^−1^cm^−1^ (A), 6,600 M^−1^cm^−1^ (C), 11,700 M^−1^cm^−1^ (G), and 8,600 M^−1^cm^−1^ (T)) of the sequence. The lysines at 3′ and 5′ end of PNAs increase their solubility. In addition to Watson Crick base pairing, the positively charged lysine amino acids of PNA improve electrostatic interactions with the negatively charged phosphate backbone of target miRs (miR-122).

### Gel shift assays

Target miR-122 was incubated with PNAs in physiological ionic strength conditions (2 mM MgCl_2_, 150 mM KCl, 10 mM sodium phosphate; pH 7.4) at 37°C in a thermal cycler (T100, Bio-Rad, Hercules, CA) for 18 hrs. The samples were then separated on a pre-cast 10% nondenaturing polyacrylamide gel using 1x tris/borate/EDTA buffer (1xTBE). The gels were run at a voltage of 120 V for 35 min. After electrophoresis, gels were stained with SYBR-Gold (Invitrogen) in 1xTBE buffer for 2 min and imaged using a Gel Doc EZ imager (Bio-Rad, Hercules, CA).

### *Immunoblotting and In situ* hybridization (ISH)

Protein samples were prepared using sample buffer and resolved using 10% SDS-PAGE under reduced conditions and transferred onto nitrocellulose membranes. Membranes were blocked using 5% BSA followed by incubation with primary antibody overnight at 4oC (dilutions-1:1000 for p-Stat3, Stat3 and Sirt1 and 1:10,000 for Gapdh). Antigen-primary antibody complexes were incubated with HRP-conjugated secondary antibodies and visualized by immunoblot luminol reagent (Thermo, USA). Images were captured and quantified using the Image Lab (Biorad, USA) software. The ISH was performed as previously described with minor modifications^19^. Briefly, the deparaffinized and rehydrated aortic sections (6 µM) were treated with proteinase K (10 µg.ml^−1^, 5 min, 37 °C). Sections were dehydrated using ethanol and incubated with pre-hybridization buffer, followed by a hybridization mixture containing either double digoxigenin (DIG)-tagged miR-204-5p probe or scrambled probe (at 20 nM for 72 hrs, 56 °C). Sections were washed with sodium saline citrate and incubated in blocking solution (5 mL PBS + 50 mg BSA + 100 µL sheep serum + 2.5 µL tween 20) for 15 min, followed by incubation with either Dylight 594-labelled goat anti-DIG antibody (working concentration-1:500, Vector laboratories) or alkaline phosphatase-conjugated anti-DIG Fab (fragment, antigen-binding; working concentration-1:500 in antibody dilution solution for 18 hrs at 4 °C). Following alkaline phosphatase-conjugated FAB binding, slides were incubated in a solution containing BCIP/NBT (5-bromo-4-chloro-3′-indolyl phosphate/nitro-blue tetrazolium) at 30 °C for 48 hrs. Slides were counterstained with 4,6-diamidino-2-phenylindole (DAPI) and dehydrated with alcohol and mounted in DPX. Images were captured by a charge-coupled device camera attached to the Zeiss microscope (Model BX61). Six images originating from at least two aortic rings were used for the quantification. The blue-purple color range was selected using Photoshop CS6, converted to grey and quantified using Image J software.

### Radio-telemetry

Carotid radio-telemetry catheters (PA-C10; Data Sciences International, St. Paul, MN) were implanted during isoflurane-induced general anesthesia (Phoenix Scientific, St. Louis, MO), as previously described^78^. Flunixin meglumine (2.5 mg/kg, Phoenix Scientific) was administered subcutaneously at the time of anesthetic induction, and 0.5% bupivacaine (Pfizer, New York, NY) was applied to the wound margin. After a 7-day recovery period, arterial pressures, heart rate, and relative locomotor activity were recorded for 10 sec every 5 min for 60 hrs (encompassing 3 dark cycles and 2 light cycles). Following those baseline recordings, L-NAME (1 mg/ml, Sigma St. Louis, MO) was administered in the drinking water, and the previous experimental protocol was repeated.

### Statistical analysis

Statistical analysis was performed using GraphPad Prism (Version 7.0). One-way analysis of variance (ANOVA) was used for multiple comparisons and posthoc analysis was performed with Tukey’s test. Independent sample t-test was used to determine the significance of difference between the two groups. The significance of the difference between two curves was analysed by using global non-linear regression. Results were expressed as mean ± s.e.m and considered significant if p values were <0.05.

## Supplementary information

Supplementary Information.

## References

[1] Gorostidi M (2011). Abnormalities in ambulatory blood pressure monitoring in hypertensive patients with diabetes. Hypertens. Res..

[2] Draman MS (2015). The importance of night-time systolic blood pressure in diabetic patients: Dublin Outcome Study. J. Hypertens..

[3] Higashi Y (2002). Circadian variation of blood pressure and endothelial function in patients with essential hypertension:a comparison of dippers and non-dippers. J. Am. Coll. Cardiol..

[4] Knudsen ST (2007). Endothelial perturbation: a link between non-dipping and retinopathy in type 2 diabetes?. J. Am. Soc. Hypertens..

[5] Lurbe E (2002). Increase in nocturnal blood pressure and progression to microalbuminuria in type 1 diabetes. N. Engl. J. Med..

[6] Verdecchia P (1994). Ambulatory blood pressure. An independent predictor of prognosis in essential hypertension. Hypertension.

[7] Durgan DJ (2016). Role of the Gut Microbiome in Obstructive Sleep Apnea-Induced Hypertension. Hypertension.

[8] Luedde M (2017). Heart failure is associated with depletion of core intestinal microbiota. ESC. Heart Fail..

[9] Marques FZ (2017). High-Fiber Diet and Acetate Supplementation Change the Gut Microbiota and Prevent the Development of Hypertension and Heart Failure in Hypertensive Mice. Circulation.

[10] Yang T (2015). Gut dysbiosis is linked to hypertension. Hypertension.

[11] Mell B (2015). Evidence for a link between gut microbiota and hypertension in the Dahl rat. Physiol. Genomics.

[12] Karbach, S. H. *et al*. Gut Microbiota Promote Angiotensin II-Induced Arterial Hypertension and Vascular Dysfunction. *J Am Heart Assoc* **5**, 10.1161/JAHA.116.003698 (2016). 10.1161/JAHA.116.003698 PMC5079031 27577581

[13] Williams MR, Stedtfeld RD, Tiedje JM, Hashsham SA (2017). MicroRNAs-Based Inter-Domain Communication between the Host and Members of the Gut Microbiome. Front. Microbiol..

[14] Zhou G, Zhou Y, Chen X (2017). New Insight into Inter-kingdom Communication: Horizontal Transfer of Mobile Small RNAs. Front. Microbiol..

[15] Bartolomaeus H (2019). Short-Chain Fatty Acid Propionate Protects From Hypertensive Cardiovascular Damage. Circulation.

[16] Hsu CN, Chang-Chien GP, Lin S, Hou CY, Tain YL (2019). Targeting on Gut Microbial Metabolite Trimethylamine-N-Oxide and Short-Chain Fatty Acid to Prevent Maternal High-Fructose-Diet-Induced Developmental Programming of Hypertension in Adult Male Offspring. Mol. Nutr. Food Res..

[17] Felizardo RJF, Watanabe IKM, Dardi P, Rossoni LV, Camara NOS (2019). The interplay among gut microbiota, hypertension and kidney diseases: The role of short-chain fatty acids. Pharmacol. Res..

[18] Bier, A. *et al*. A High Salt Diet Modulates the Gut Microbiota and Short Chain Fatty Acids Production in a Salt-Sensitive Hypertension Rat Model. *Nutrients* **10**, 10.3390/nu10091154 (2018). 10.3390/nu10091154 PMC6164908 30142973

[19] Vikram A (2016). Vascular microRNA-204 is remotely governed by the microbiome and impairs endothelium-dependent vasorelaxation by downregulating Sirtuin1. Nat. Commun..

[20] Courboulin A (2011). Role for miR-204 in human pulmonary arterial hypertension. J. Exp. Med..

[21] Yu C (2018). LncRNA TUG1 sponges miR-204-5p to promote osteoblast differentiation through upregulating Runx2 in aortic valve calcification. Cardiovasc. Res..

[22] Zheng X (2009). Association of SLC34A2 variation and sodium-lithium countertransport activity in humans and baboons. Am. J. Hypertens..

[23] Marzano F (2018). Pilot study on circulating miRNA signature in children with obesity born small for gestational age and appropriate for gestational age. Pediatr. Obes..

[24] Wang R (2015). Elevated circulating microRNA-122 is associated with obesity and insulin resistance in young adults. Eur. J. Endocrinol..

[25] Prats-Puig A (2013). Changes in circulating microRNAs are associated with childhood obesity. J. Clin. Endocrinol. Metab..

[26] Ortega FJ (2013). Targeting the circulating microRNA signature of obesity. Clin. Chem..

[27] Gao W (2012). Plasma levels of lipometabolism-related miR-122 and miR-370 are increased in patients with hyperlipidemia and associated with coronary artery disease. Lipids Health Dis..

[28] Li XD (2017). Elevated plasma miRNA-122, -140-3p, -720, -2861, and -3149 during early period of acute coronary syndrome are derived from peripheral blood mononuclear cells. PLos One.

[29] Li X (2015). Plasma miR-122 and miR-3149 Potentially Novel Biomarkers for Acute Coronary Syndrome. PLoS One.

[30] D’Alessandra Y (2010). Circulating microRNAs are new and sensitive biomarkers of myocardial infarction. Eur. Heart J..

[31] Willeit P, Skroblin P, Kiechl S, Fernandez-Hernando C, Mayr M (2016). Liver microRNAs: potential mediators and biomarkers for metabolic and cardiovascular disease?. Eur. Heart J..

[32] Xu G, Chen J, Jing G, Shalev A (2013). Thioredoxin-interacting protein regulates insulin transcription through microRNA-204. Nat. Med..

[33] Cheng CW (2017). Fasting-Mimicking Diet Promotes Ngn3-Driven beta-Cell Regeneration to Reverse Diabetes. Cell.

[34] Chugh SN, Dabla S, Jain V, Chugh K, Sen J (2010). Evaluation of endothelial function and effect of glycemic control (excellent vs. poor / fair control) on endothelial function in uncontrolled type 2 diabetes mellitus. J. Assoc. Physicians India.

[35] Zhong JC (2007). Apelin modulates aortic vascular tone via endothelial nitric oxide synthase phosphorylation pathway in diabetic mice. Cardiovasc. Res..

[36] Zhong JC (2007). The novel peptide apelin regulates intrarenal artery tone in diabetic mice. Regul. Pept..

[37] Perk G, Mekler J, Ben Ishay D, Bursztyn M (2002). Non-dipping in diabetic patients: insights from the siesta. J. Hum. Hypertens..

[38] Surendran P (2016). Trans-ancestry meta-analyses identify rare and common variants associated with blood pressure and hypertension. Nat. Genet..

[39] Liu C (2016). Meta-analysis identifies common and rare variants influencing blood pressure and overlapping with metabolic trait loci. Nat. Genet..

[40] Ehret GB (2016). The genetics of blood pressure regulation and its target organs from association studies in 342,415 individuals. Nat. Genet..

[41] Park Y, Subar AF, Hollenbeck A, Schatzkin A (2011). Dietary fiber intake and mortality in the NIH-AARP diet and health study. Arch. Intern. Med..

[42] Jo S (2018). miR-204 Controls Glucagon-Like Peptide 1 Receptor Expression and Agonist Function. Diabetes.

[43] Klein D (2013). MicroRNA expression in alpha and beta cells of human pancreatic islets. Plos One.

[44] Li Volti G (2011). Effect of silibinin on endothelial dysfunction and ADMA levels in obese diabetic mice. Cardiovasc. Diabetol..

[45] Ceriello A (2008). Oscillating glucose is more deleterious to endothelial function and oxidative stress than mean glucose in normal and type 2 diabetic patients. Diabetes.

[46] Yu, F. *et al*. Abnormal gut microbiota composition contributes to the development of type 2 diabetes mellitus in db/db mice. *Aging (Albany NY)* **11**, 10.18632/aging.102469 (2019). 10.18632/aging.102469 PMC6914402 31760385

[47] Beli E (2018). Restructuring of the Gut Microbiome by Intermittent Fasting Prevents Retinopathy and Prolongs Survival in db/db Mice. Diabetes.

[48] Chen C (2018). Modulation of gut microbiota by mulberry fruit polysaccharide treatment of obese diabetic db/db mice. Food Funct..

[49] Li, X. W. *et al*. Effects of Rich-Polyphenols Extract of Dendrobium loddigesii on Anti-Diabetic, Anti-Inflammatory, Anti-Oxidant, and Gut Microbiota Modulation in db/db Mice. *Molecules* **23**, 10.3390/molecules23123245 (2018). 10.3390/molecules23123245 PMC6320866 30544624

[50] Hui, S. *et al*. Capsaicin Improves Glucose Tolerance and Insulin Sensitivity Through Modulation of the Gut Microbiota-Bile Acid-FXR Axis in Type 2 Diabetic db/db Mice. *Mol Nutr Food Res*, e1900608, 10.1002/mnfr.201900608 (2019). 10.1002/mnfr.201900608 31539192

[51] Tatemoto K (2001). The novel peptide apelin lowers blood pressure via a nitric oxide-dependent mechanism. Regul. Pept..

[52] Cox CM, D’Agostino SL, Miller MK, Heimark RL, Krieg PA (2006). Apelin, the ligand for the endothelial G-protein-coupled receptor, APJ, is a potent angiogenic factor required for normal vascular development of the frog embryo. Dev. Biol..

[53] Iliopoulos D, Drosatos K, Hiyama Y, Goldberg IJ, Zannis VI (2010). MicroRNA-370 controls the expression of microRNA-122 and Cpt1alpha and affects lipid metabolism. J. Lipid Res..

[54] Yoshikawa T (2012). Silencing of microRNA-122 enhances interferon-alpha signaling in the liver through regulating SOCS3 promoter methylation. Sci. Rep..

[55] Lu X (2019). Circ_1639 induces cells inflammation responses by sponging miR-122 and regulating TNFRSF13C expression in alcoholic liver disease. Toxicol. Lett..

[56] Tang Y (2017). CCL2 is Upregulated by Decreased miR-122 Expression in Iron-Overload-Induced Hepatic Inflammation. Cell Physiol. Biochem..

[57] Noh K (2017). miR-122-SOCS1-JAK2 axis regulates allergic inflammation and allergic inflammation-promoted cellular interactions. Oncotarget.

[58] Li M (2017). The hepatocyte-specific HNF4alpha/miR-122 pathway contributes to iron overload-mediated hepatic inflammation. Blood.

[59] Thakral S, Ghoshal K (2015). miR-122 is a unique molecule with great potential in diagnosis, prognosis of liver disease, and therapy both as miRNA mimic and antimir. Curr. Gene Ther..

[60] Ward J (2014). Circulating microRNA profiles in human patients with acetaminophen hepatotoxicity or ischemic hepatitis. Proc. Natl Acad. Sci. USA.

[61] Antoine DJ (2013). Mechanistic biomarkers provide early and sensitive detection of acetaminophen-induced acute liver injury at first presentation to hospital. Hepatology.

[62] Xu J (2011). Circulating microRNAs, miR-21, miR-122, and miR-223, in patients with hepatocellular carcinoma or chronic hepatitis. Mol. Carcinog..

[63] Della Mea P (2005). Adiponectin, insulin resistance, and left ventricular structure in dipper and nondipper essential hypertensive patients. Am. J. Hypertens..

[64] Dharmashankar K, Widlansky ME (2010). Vascular endothelial function and hypertension: insights and directions. Curr. Hypertens. Rep..

[65] Bodary PF (2007). Leptin regulates neointima formation after arterial injury through mechanisms independent of blood pressure and the leptin receptor/STAT3 signaling pathways involved in energy balance. Arterioscler. Thromb. Vasc. Biol..

[66] Moriyama T, Oka K, Ueda H, Imai E (2004). Nilvadipine attenuates mesangial expansion and glomerular hypertrophy in diabetic db/db mice, a model for type 2 diabetes. Clin. Exp. Nephrol..

[67] Bagi Z (2005). Type 2 diabetic mice have increased arteriolar tone and blood pressure: enhanced release of COX-2-derived constrictor prostaglandins. Arterioscler. Thromb. Vasc. Biol..

[68] Su W (2008). Hypertension and disrupted blood pressure circadian rhythm in type 2 diabetic db/db mice. Am. J. Physiol. Heart Circ. Physiol.

[69] Senador D, Kanakamedala K, Irigoyen MC, Morris M, Elased KM (2009). Cardiovascular and autonomic phenotype of db/db diabetic mice. Exp. Physiol..

[70] Corsten MF (2010). Circulating MicroRNA-208b and MicroRNA-499 reflect myocardial damage in cardiovascular disease. Circ. Cardiovasc. Genet..

[71] Novak J, Olejnickova V, Tkacova N, Santulli G (2015). Mechanistic Role of MicroRNAs in Coupling Lipid Metabolism and Atherosclerosis. Adv. Exp. Med. Biol..

[72] Pluznick JL (2013). Olfactory receptor responding to gut microbiota-derived signals plays a role in renin secretion and blood pressure regulation. Proc. Natl Acad. Sci. USA.

[73] Miura K (2004). Relation of vegetable, fruit, and meat intake to 7-year blood pressure change in middle-aged men: the Chicago Western Electric Study. Am. J. Epidemiol..

[74] Ascherio A (1996). Prospective study of nutritional factors, blood pressure, and hypertension among US women. Hypertension.

[75] Petry CJ, Ong KK, Hughes IA, Acerini CL, Dunger DB (2017). Associations between bacterial infections and blood pressure in pregnancy. Pregnancy Hypertens..

[76] Wang Z (2011). Gut flora metabolism of phosphatidylcholine promotes cardiovascular disease. Nature.

[77] Tahmasbi Rad A (2019). A universal discoidal nanoplatform for the intracellular delivery of PNAs. Nanoscale.

[78] Roghair RD (2009). Vascular nitric oxide and superoxide anion contribute to sex-specific programmed cardiovascular physiology in mice. Am. J. Physiol. Regul. Integr. Comp. Physiol.

