# Supplementary material for: Microbiota-governed microRNA-204 impairs endothelial function and blood pressure decline during inactivity in db/db mice
Source: Sci Rep. 2020 Jun 22;10:10065. doi: 10.1038/s41598-020-66786-0 (PMC7308358; doi:10.1038/s41598-020-66786-0)
Supplement: Supplementary file 1 — Supplementary Information. [file 41598_2020_66786_MOESM1_ESM.docx]

**Supplementary Information**

**Microbiota-governed microRNA-204 impairs endothelial function and arterial decline in blood pressure at rest in db/db mice**

Ravinder Reddy Gaddam^1^, Veronica Peotta Jacobsen^2^, Young-Rae Kim^1^, Mohanad Gabani^1^, Julia S. Jacobs^1^, Karishma Dhuri^3^, Santosh Kumar^1^, Modar Kassan^1^, Qiuxia Li^1^, Raman Bahal^3^, Robert Roghair^2^, Kaikobad Irani^1^, Ajit Vikram^1^*

^1^Department of Internal Medicine, Carver College of Medicine University of Iowa, Iowa City, IA-52242, USA.

^2^Department of Paediatrics, Carver College of Medicine University of Iowa, Iowa City, IA-52242, USA.

^3^Department of Pharmaceutical Sciences, University of Connecticut, Connecticut, USA.

* To whom correspondence should be addressed.

Tel: 001 (319)3352153;

Email: [ajit-vikram@uiowa.edu](mailto:ajit-vikram@uiowa.edu)

**
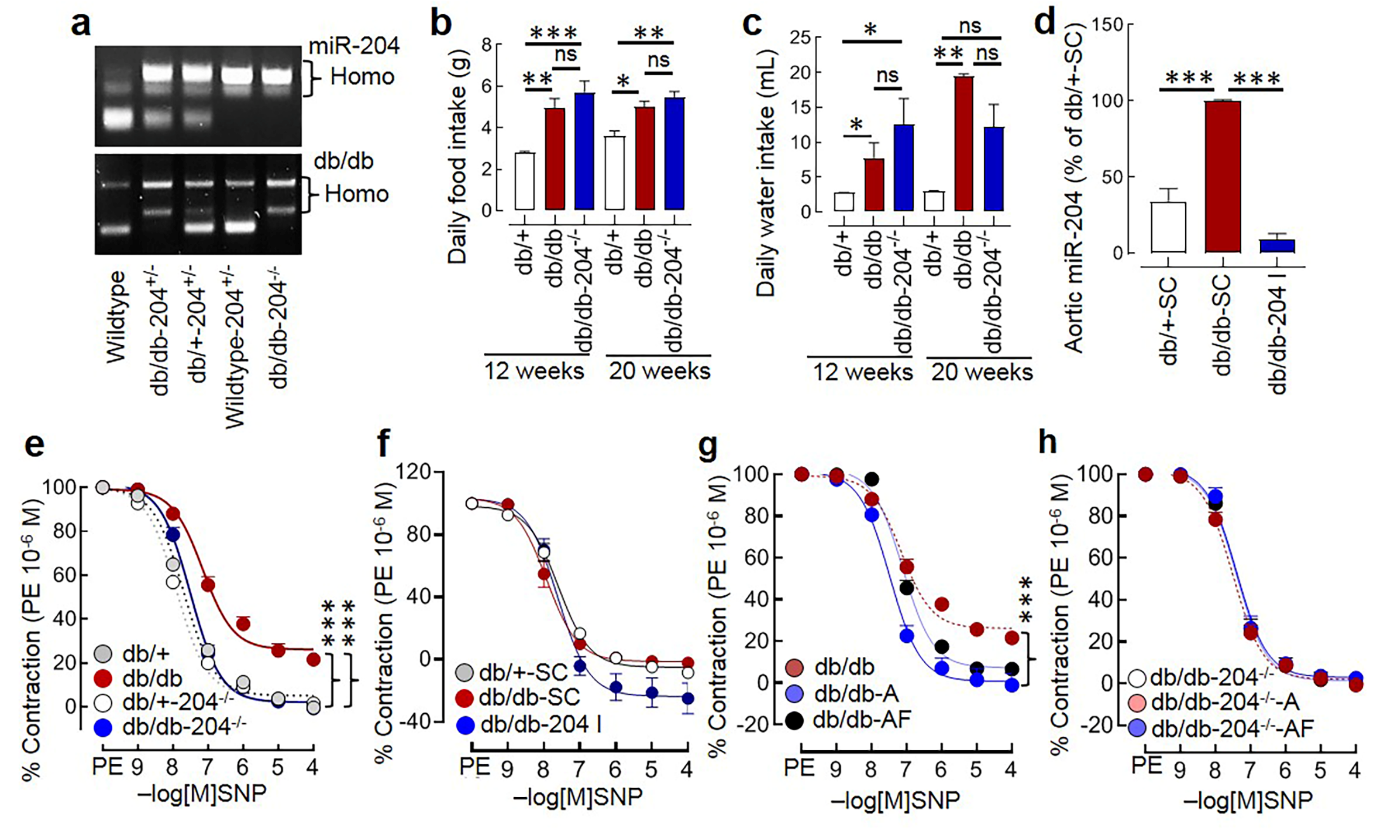
**

**Supplementary Figure S1.** **a**) Generation of db/+, db/db, and db/db-204^-/-^ mice. The full-length gel is presented in Supplementary Figure S7. **b & c**) Daily food (b) and water intake (c) in db/+, db/db, and db/db-204^-/-^ mice at 12 weeks and 20 weeks of age. n=4-9. **d**) Aortic miR-204 in the *ex vivo* transfected aortas in Fig. 1h. n=3-4. **e)** The SNP-induced vascular relaxation of the aortic rings isolated from db/+, db/+-204^-/-^, db/db, and db/db-204^-/-^ mice. db/+: n=13(4), db/+-204^-/-^: n=11(3), db/db: n=21(7), db/db-204^-/-^: n=12(4). **f)** The SNP-induced vascular relaxation of the aortic rings isolated from db/+ or db/db mice transfected with either SC or miR-204 I. db/+-SC: n=5(3), db/db-SC: n=9(4), db/db-204 I: n=5(3). **g & h)** The SNP-mediated vascular relaxation of the aortic rings isolated from db/db (g) and db/db-204^-/-^ (h) mice after antibiotics treatment (A) and microbial recolonization (F). db/db: n=21(7), db/db-A: n=9(5), db/db-AF: n=15(5), db/db-204^-/-^: n=12(4), db/db-204^-/-^-A: n=5(4), and db/db-204^-/-^-AF: n=18(8). The experimental replicate is shown as "n(N)" where 'n' represents the number of aortic rings and 'N' represents the number of mice. A one-way analysis of variance (ANOVA) followed by Tukey’s test was performed for figures b-d. The significance of the difference between two curves (Fig. e-h) was analyzed by using global non-linear regression. ns>0.05, *p< 0.05, **p< 0.01 and ***p< 0.001 vs. indicated group. The data shown as mean and error bar represents s.e.m. PE, phenylephrine; SNP, sodium nitroprusside.

**
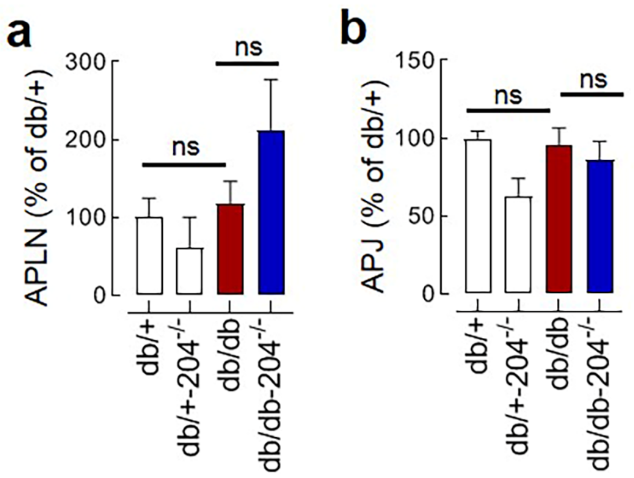
**

**Supplementary Figure S2.** **a & b**) Vascular expression of APLN (a) and APJ (b) in the db/+, db/+-204^-/-^, db/db, and db/db-204^-/-^ mice. db/+: n=5, db/+-204^-/-^: n=4, db/db: n=9, db/db-204^-/-^: n=6. ns>0.05 vs. indicated group. The data shown as mean and error bar represents s.e.m. The significance of the difference between groups was performed by ANOVA followed by Tukey’s test.


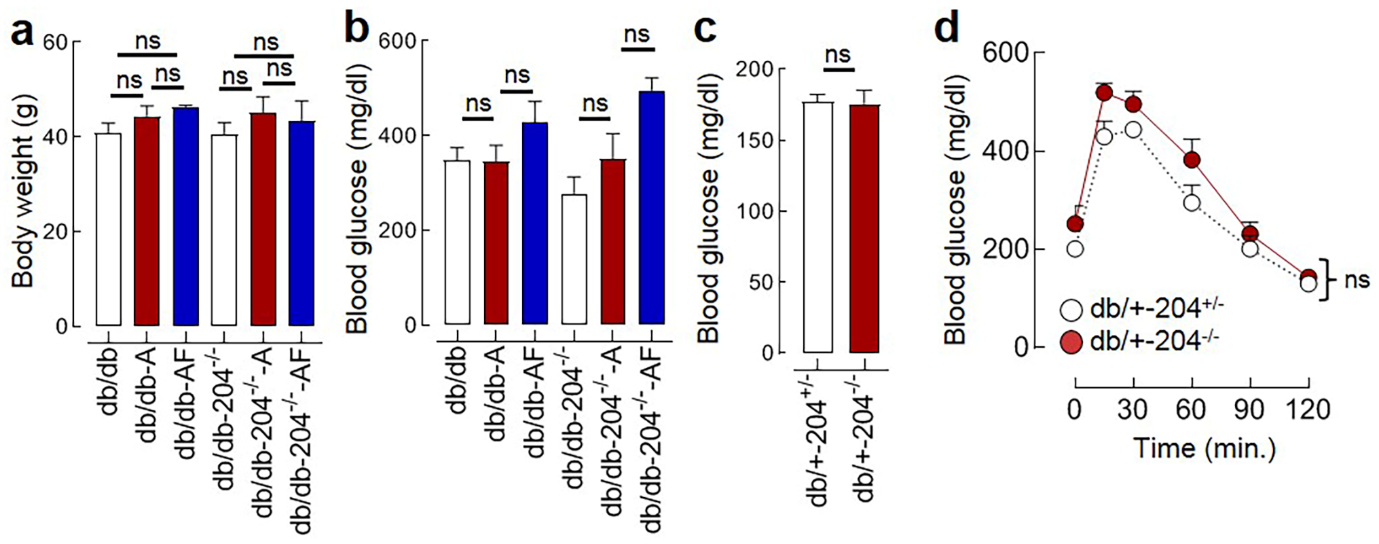


**Supplementary Figure S3.** **a & b)** Body weight (a) and blood glucose levels (b) in db/db and db/db-204^-/-^ mice after antibiotics and FMT. db/db: n=15, db/db-A: n=9, db/db-AF: n=5, db/db-204^-/-^: n=11, db/db-204^-/-^-A: n=7, db/db-204^-/-^-AF: n=6. **c & d)** The fasting blood glucose levels (c) and glucose tolerance test (GTT) (d) in db/+-204^+/-^ and db/+-204^-/-^ mice. Glucose (2 g/kg, oral) was administered at 0 min, and blood glucose levels were measured at the designated time points. db/+-204^+/-^: n=5, and db/+-204^-/-^: n=5. ns>0.05 vs. indicated group. The data are shown as mean, and error bar represents s.e.m. The significance of the difference between groups (in Fig. a & b) was performed by ANOVA followed by Tukey’s test. An independent sample t-test was used to compare two groups in Fig. c. The significance of difference between two curves (in Fig. d) was analyzed by using global non-linear regression.


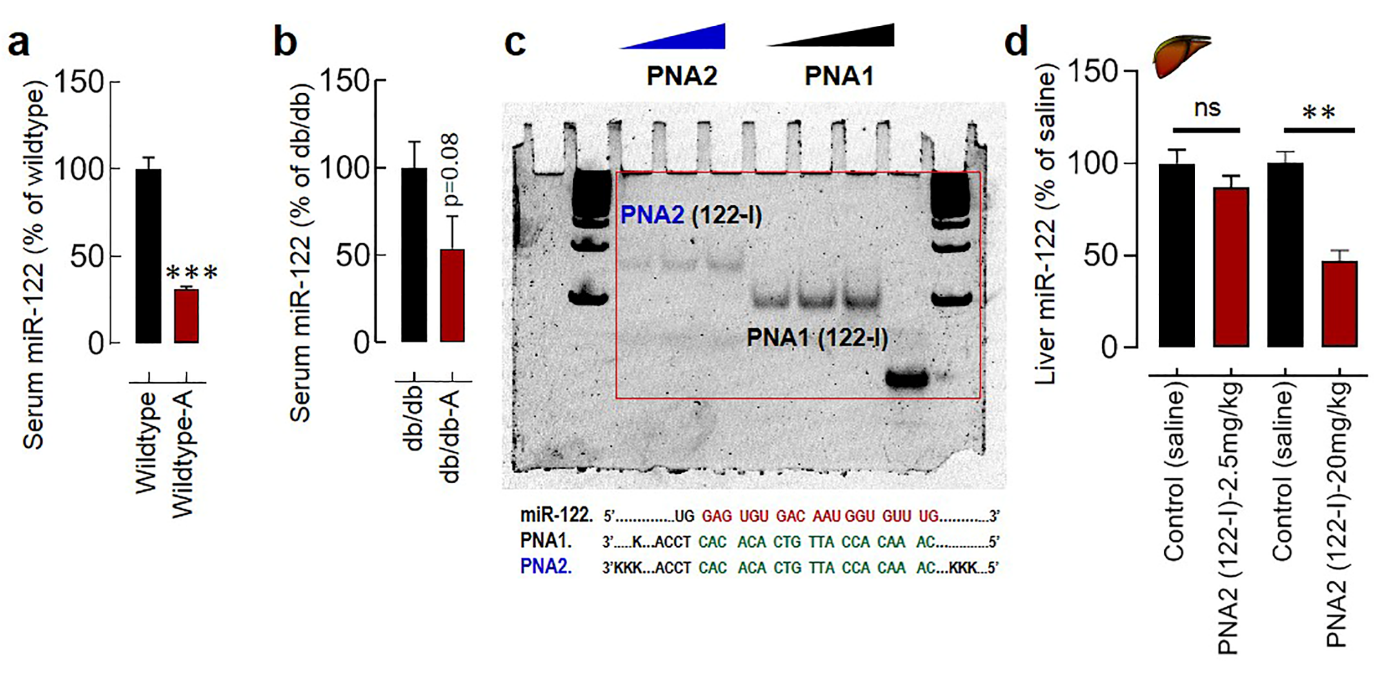


**Supplementary Figure S4.** **a & b)** The circulating miR-122 in wildtype (a) and db/db (b) mice after antibiotics treatment. n=4-5. **c)** PNA2 with 3 lysines is more effective in inhibiting miR-122. **d**) *In vivo* delivery of PNA-122-I (PNA2) at 2.5 or 20mg/kg/day for 5 days decreases liver miR-122 expression. The control group received saline. ns>0.05, **p < 0.01, and ***p < 0.001 vs. indicated group. The data are shown as mean, and error bar represents s.e.m. An independent sample t-test was used.

**
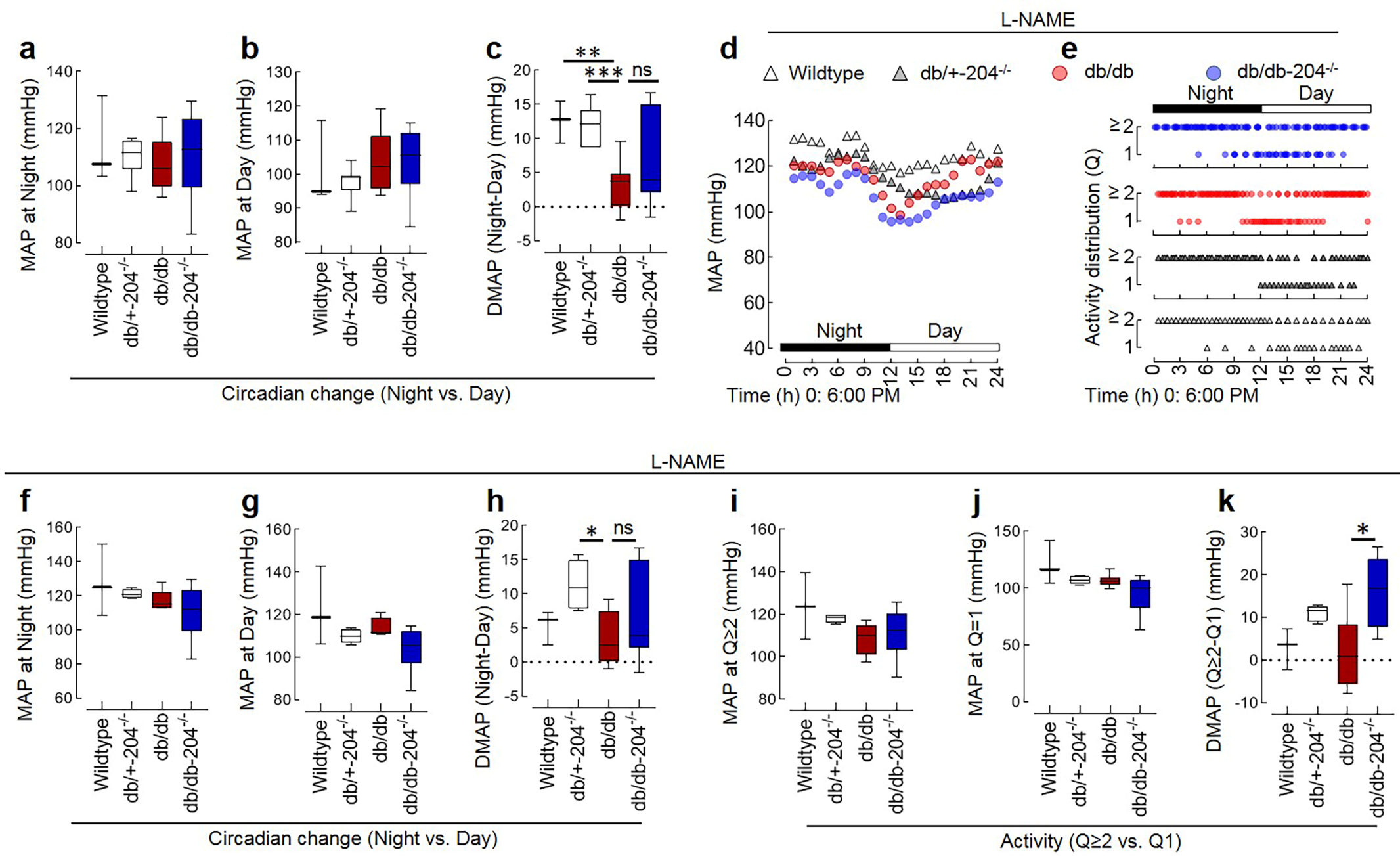
**

**Supplementary Figure S5.** **The MAP in the Wildtype, db/+-204^-/-^, db/db, and db/db-204^-/-^ mice. a-c)** The MAP during the night (a), day (b), and circadian decline in the MAP (c). Wildtype: n=3, db/+-204^-/-^: n=7, db/db: n=7, db/db-204^-/-^: n=6. **d**) A representative curve of 24 hr (day and night) telemetric recording of the MAP in the Wildtype, db/+-204^-/-^, db/db, and db/db-204^-/-^ mice after L-NAME. **e)** The mouse locomotor activity distribution in quartiles after L-NAME. The 'y' axis shows hourly activity quartile. 1; 0-25%, ≥2; 25-100%. The 'x' axis shows time (24 h) beginning at 6:00 PM. **f-h**) The MAP during the night (f), day (g), and circadian decline in the MAP after L-NAME (h). Wildtype: n=3, db/+-204^-/-^: n=4, db/db: n=6, db/db-204^-/-^: n=6. **i-k**) The MAP during activity quartile ≥2 (i), activity quartile 1 (j) and a difference in the MAP during activity quartile ≥2 and activity quartile 1 (k) after L-NAME. Wildtype: n=3, db/+-204^-/-^: n=4, db/db: n=6, db/db-204^-/-^: n=6. ns>0.05, *p<0.05; **p<0.01; ***p<0.001 vs. indicated group. In box-and-whisker plots, whiskers show minima and maxima and the central line indicates median. The significance of the difference between groups was performed by ANOVA followed by Tukey’s test.


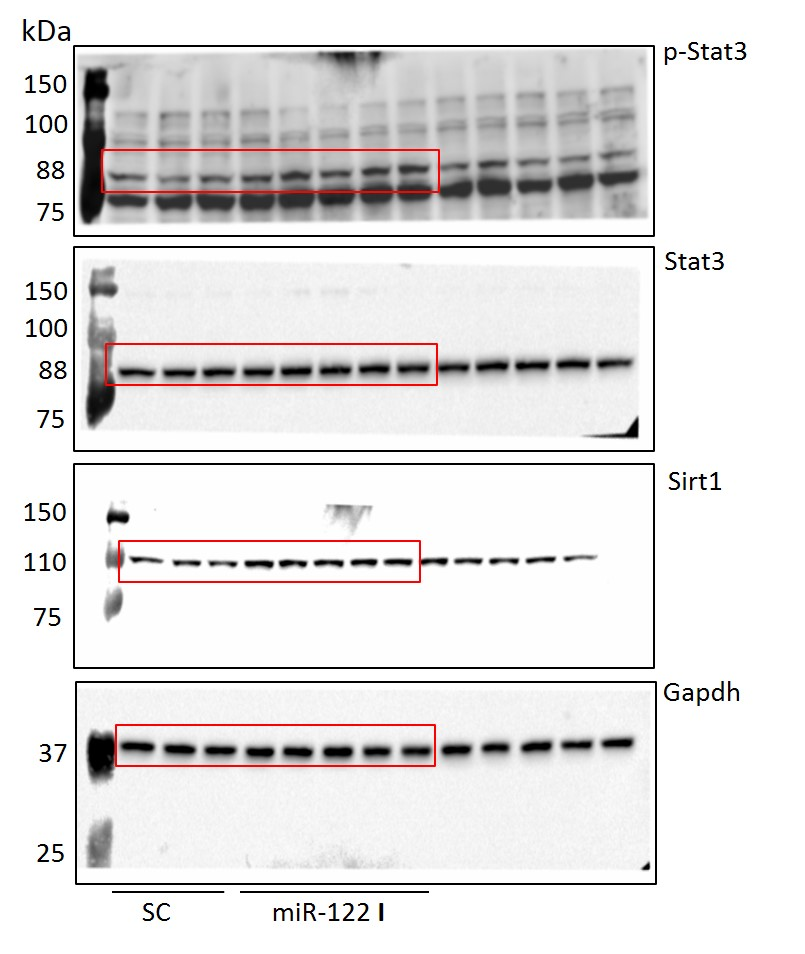


**Supplementary Figure S6.** Uncropped western blots with molecular weight marker for p-Stat3, Stat3, Sirt1 and Gapdh.


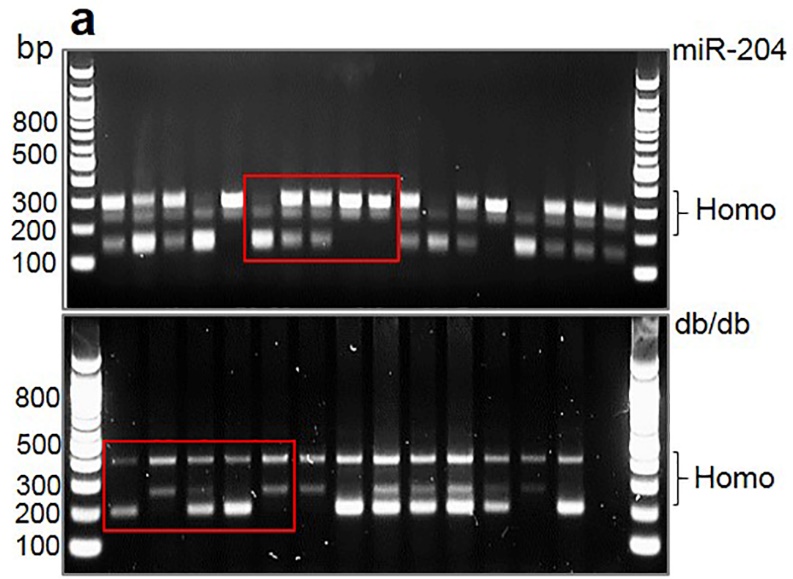


**Supplementary Figure S7.** Uncropped agarose gels with base pair marker for generation of db/+, db/db, and db/db-204^-/-^ mice.

| **Supplementary Table S1.** The sequence of primers, mature microRNAs, detection probes, and microRNA-mimic/inhibitor used in the study. | | | |
| --- | --- | --- | --- |
| **mRNA** | | **Primer sequence** | |
|  |  | **Forward** | **Reverse** |
| GAPDH (mouse) | | 5’-GGC AAA TTC AAC GGC ACA GT-3’ | 5’-CGC TCC TGG AAG ATG GTG AT-3’ |
| 16S (bacterial) | | 5’-ACT CCT ACG GGA GGC AGC AGT-3’ | 5’-GTA TTA CCG CGG CTG CTG GCA C-3’ |
| APLN | | 5'-CCT TGA CTG CAG TTT GTG GA-3' | 5'-CTC GAA GTT CTG GGC TTC AC-3' |
| APJ | | 5'-CCA CCT GGT GAA GAC TCT CTA CA-3' | 5'-CTG ACG TAA CTG ATG CAG GTG-3' |
| **microRNA** | | **Mature microRNA sequence** | |
| microRNA-204-5p | | 5'-UUC CCU UUG UCA UCC UAU GCC U-3' | |
| microRNA0122-5p | | 5'-UGG AGU GUG ACA AUG GUG UUU G-3' | |
| RNU6 | | 5'-CGC AAG GAU GAC ACG CAA AUU CGU GAA GCG UUC CAU AUU UUU-3' | |
| **microRNA** | | **Primer Sequence** | |
| microRNA-204-5p | | 5’-CGC TTC CCT TTG TCA TCC TA-3’ | |
| microRNA-122-5p | | 5’-TGG AGT GTG ACA ATG GTG TTT G-3’ | |
| RNU6 | | 5’-GCA AAT TCG TGA AGC GTT CC-3’ | |
| **Modulators** | | **Sequence** |  |
| Scrambled control | | 5’-ACG TCT ATA CGC CCA- 3’ | |
| microRNA-204-inhibitor | | 5’-AGG ATG ACA AAG GGA-3’ | |
| microRNA-204 ISH probe | | 5’-Dig-N-AGG CAT AGG ATG ACA AAG GGA A-N-Dig-3’ | |
| The scrambled ISH probe | | 5’-Dig-N-GTG TAA CAC GTC TAT ACG CCC A-N-Dig-3’ | |
| **Genotyping** | | **Primer Sequence** | |
|  |  | **Forward** | **Reverse** |
| miR-204 | | 5’-AAT GCTGGT CAG TGG CTA AGA-TGC-3’ | 5’-AGG AAA GTT ATG GGC TCA ATG ATG G-3’ |
| Lepr (db/db) | inner | 5’-TAT TAG AAG ATG TTT TGA TGG AGG G-3’ | 5’-GTC ATT CAA ACC ATA GTT TAG GTT TGT TAA-3’ |
|  | outer | 5’-AGG ATA CAA TAC AAG AAC AAA AAG CCT G-3’ | 5’-ATG CAG AGT CCA TGA ATA TCA ACT TTA A-3’ |
